# Surface stress of graphene layers supported on soft substrate

**DOI:** 10.1038/srep25653

**Published:** 2016-05-11

**Authors:** Feng Du, Jianyong Huang, Huiling Duan, Chunyang Xiong, Jianxiang Wang

**Affiliations:** 1 State Key Laboratory for Turbulence and Complex System, and Department of Mechanics and Engineering Science, College of Engineering, Peking University, Beijing 100871, China; 2 Department of Biomedical Engineering, Duke University, Durham, NC, 27708, USA; 3 CAPT-HEDPS, and IFSA Collaborative Innovation Center of MoE, Peking University, Beijing 100871, China; 4 Academy for Advanced Interdisciplinary Studies, Peking University, Beijing 100871, China

## Abstract

We obtain the surface stress of a single layer and multilayers of graphene supported on silicone substrates by measuring the deformation of the graphene-covered substrates induced by the surface tension of liquid droplets together with the Neumann’s triangle concept. We find that the surface stress of the graphene-covered substrate is significant larger than that of the bare substrate, and it increases with increasing graphene layers, and finally reaches a constant value of about 120 mN/m on three and more layers of graphene. This work demonstrates that the apparent surface stress of graphene-substrate systems can be tuned by the substrate and the graphene layers. The surface stress and the tuning effect of the substrate on it may have applications in design and characterization of graphene-based ultra-sensitive sensors and other devices. Moreover, the method may also be used to measure the surface stress of other ultrathin films supported on soft substrates.

Surface stress of solids, which connects the macroscopic properties of solids and the chemical bonding state at a surface or an interface^1^,^2^,^3^, plays an important role in many surface phenomena such as elastic moduli of nanoscale materials^4^,^5^,^6^, surface reconstruction of metals^7^,^8^, shape transitions of nanoparticles^9^, surface diffusion, epitaxial growth^10^, self-assembled domain patterns^11^, deformation of nanoporous materials^12^,^13^, bending of layered cantilevers^14^,^15^, contacts of soft matters^16^, and stiffening of solids by surface stress^17^,^18^. Another important surface property is surface energy, and the relation between surface stress and surface energy is elaborated in many papers^2^,^3^,^4^. Graphene is a typical two-dimensional or surface material^19^,^20^. In many applications, graphene layers are supported on a substrate^21^, such as graphene-based electrodes^22^,^23^, solar cells^24^, and artificial actuators^23^. Thus, the surface properties of graphene layers and the effect of the underlying substrates on their surface properties have significant implications in design of graphene-based devices, and thus attracted much attention^25^,^26^,^27^,^28^,^29^,^30^. For example, graphene layers supported on soft substrates, by converting the deformation of the layered system under lateral loading into electronic signals, can make ultra-sensitive flexible tactile sensors attaining the lowest human pressure perception^31^,^32^,^33^. Surface stress and interface stress can be principal factors in determining the deformation of nanoscale films and multilayers^4^,^5^,^15^,^34^,^35^, and thus, control of their effect is regarded as “the core of design and construction of ultra-sensitive mechanical sensors”^36^. Mechanical deformation also affects the electronic structure of graphene^37^,^38^. However, to our knowledge, there is no report about the apparent surface stress of graphene supported on substrates. Moreover, graphene is the thinnest material ever found^39^. When combining it with a substrate, one may study the relative contribution of the atomic layers of the substrate beneath the thin membrane to the apparent surface stress of the membrane-substrate composite system.

Nevertheless, the effects of surface stresses are not notable in normal conditions, and therefore, direct experimental measurement of the value of surface stress of solids has remained a challenge for a long time. The reported surface stress of solids is mostly based on theoretical calculations^2^,^35^. The available surface stress that has been obtained from experiments is confined to soft polymers, such as silicone^16^,^40^,^41^, which has an elasto-capillary length *γ*_*s*_/*E* of several tens of microns, where *γ*_*s*_ is the surface stress and *E* is the Young’s modulus of the solid^42^. Recent research reveals that, when a liquid droplet contacts a soft elastic substrate, a deformed microscopic region will emerge at the vicinity of the contact line, and the shape of this region can be used to calculate the surface stress directly^40^,^41^.

## Results

### Deformation influenced by graphene

**Figure 1:**
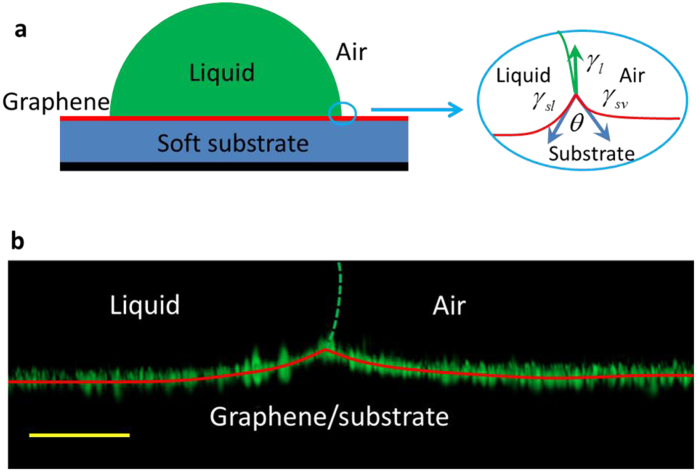
Deformation of a soft substrate covered by graphene. (**a**) Schema of a sessile drop on a soft substrate covered by graphene. (**b**) Locations of embedded fluorescent beads when a glycerol droplet stands on a 30 μm thick PDMS substrate covered by one layer of graphene (1LG). The red continuous line is the fitting curve of the surface profile from the centre of the fluorescent beads, while the green dashed line is the surface of the glycerol droplet. The scale bar is 10 μm.

**Figure 2:**
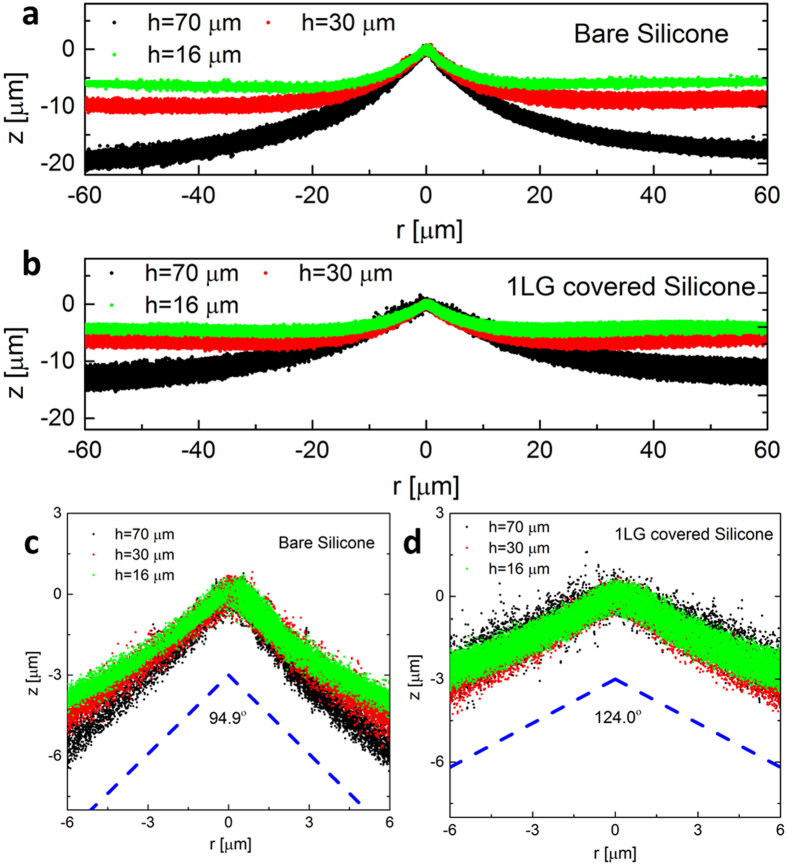
Surface profiles near the contact line. (**a**,**b**) are the deformation profiles induced by a glycerol droplet on a bare silicone substrate of PDMS and a substrate covered by one layer of graphene (1LG), respectively. The cusp regions of the substrates with different thicknesses (shown in different colours) are aligned to the origin point (z = 0). (**c**,**d**) are the close-ups of the cusp regions in (**a**,**b**), with the dashed lines showing the extracted cusp angles.

In this Article, we investigate the apparent surface stress of graphene supported on silicone substrates (Fig. 1), by extending the method recently developed by Style *et al.*^40^, who measured the surface stress of pure silicone based on the deformation of the substrate induced by a liquid droplet. Here, graphene layers were supported on two kinds of soft silicone, i.e., one type of polydimethylsiloxane with a high softness (PDMS) and another type of highly elastic silicone gel of CY52-276A/B (CY52-276) (See Methods for details). Two kinds of liquid, i.e., glycerol and liquid paraffin, were used to induce the deformation of the substrates by the surface tension (Fig. 1a) (Supplementary information). We embedded fluorescent beads on the surface of the soft silicone substrate to trace the deformation. Figure 1b shows the positions of the embedded fluorescent beads located on the surface of a silicone substrate of PDMS that is covered by one layer of graphene and deformed by a glycerol droplet, where the surface profile can be obtained by extracting the centre of each fluorescent bead^40^,^43^. Figure 2(a,b) show the extracted surface profiles of bare and one layer graphene-covered PDMS substrates of different thicknesses under glycerol droplets (See Supplementary information for the results of liquid paraffin). The height of the profile is determined by the vertical component of the liquid surface tension^43^,^44^,^45^.

When focusing on the profile of the contact line region in Fig. 2(a,b), and, in particular, the close-up of this region in Fig. 2(c,d), we find that for a given substrate and liquid, all the scattered points collapse into a cusp region, as discovered for pure silicone substrates^40^, irrespective of the substrate thickness and the existence of graphene. Theoretically, the thickness only influences the deformation of the substrate that is many micrometers away from the contact line, and does not influence the angle of the cusp which can be determined from a region near the contact line. Using the method of Style *et al.*^40^, we can calculate the angle of the cusp. Both of the two types of liquids cause a larger cusp angle on the graphene-covered substrate than that on the bare counterpart (Supplementary information). For example, the cusp angle of the bare PDMS substrate caused by a glycerol droplet is 94.9° ± 2.4°, whereas that of the substrate covered by one layer of graphene increases to 124.0° ± 0.3°.

### Model of deformation with graphene membrane coverage

**Figure 3:**
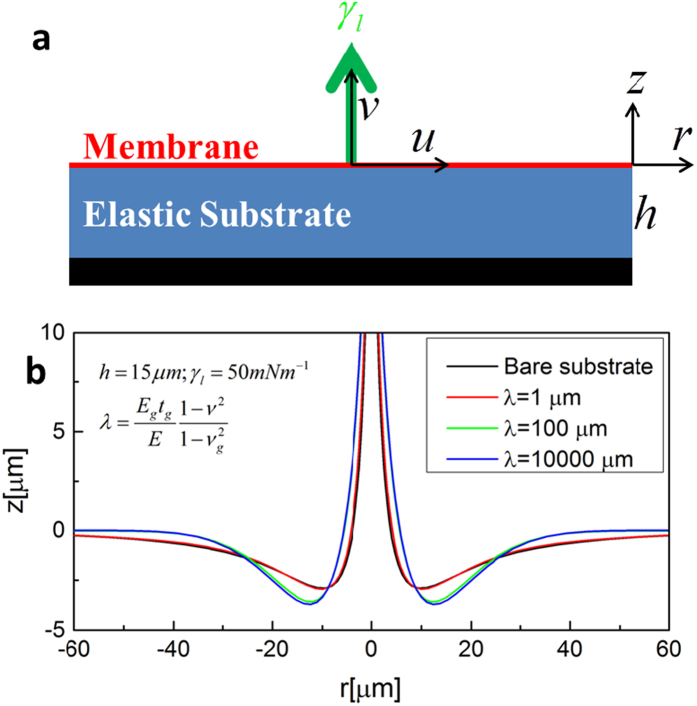
(**a**) An out-of-plane force exerted on an elastic substrate covered by a membrane. (**b**) Deformation profile of the membrane-covered elastic substrate in the absence of surface stress. The thickness of the substrate is 15 μm and the liquid tension *γ*_*l*_ is 50 mN/m. The equivalent thickness *λ* of the membrane varies from 1 μm to 10^4^ μm.

The similar deformation profiles and cusp shapes caused by liquid droplets near the contact lines on the bare and graphene-covered substrates imply that the same rule governs the deformation. Because graphene is the stiffest material ever found^46^,^47^, the increase in the cusp angle of the graphene-covered substrate may be a consequence of the joint effect of the mechanical reinforcement of graphene and the surface stress. Therefore, we generalize the model of Jerison *et al.*^43^ for a pure substrate to a substrate covered by a graphene membrane to single out the effect of the surface stress (schema shown in Fig. 3a). As the deformation caused by a liquid droplet is significantly smaller than the contact radius of the droplet on the substrate, we employ a two-dimensional elasticity model to analyze the substrate deformation^43^. Introduce the Airy stress function *ϕ* that satisfies the biharmonic equation:


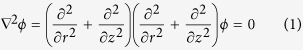


where *r* and *z* represent the radial and vertical coordinates from the position of the contact line on the surface of the substrate, respectively. The stress and strain of the substrate can be found in ref. 48. Graphene is modeled as a membrane for its thickness is ultra-small compared to other length scales of the system. The deformation of graphene can be described by^49^


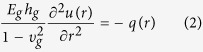


where *u*(*r*) is the displacement in the horizontal direction; *q*(*r*) is the interface stress between graphene and the substrate; *E_g_*, *h*_*g*_ and *v*_*g*_ are the Young’s modulus, the thickness, and the Poisson’s ratio of graphene, respectively. Using the continuity conditions of the displacements and stresses at the interface of graphene and the substrate, this problem can be solved analytically based on the Fourier transform procedure (Supplementary information and ref. 48). Figure 3b displays the profiles of the deformed surfaces calculated from the above theory. Without considering the surface stress of the solid, the displacement is singular at the contact line no matter how stiff the membrane is, as characterized by the classical Boussinesq solution. Thus, the mechanical reinforcement of the graphene membrane shows little effect on the deformation pattern.

### Surface stress of substrate covered by one layer of graphene

Following the method developed in ref. 40, which is based on the Neumann’s triangle concept that the surface stress of the solid, the interface stress between the liquid and solid, and the surface tension of the liquid are in equilibrium at the contact point, we extract the surface/interface stress through


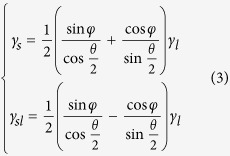


where *γ*_*sl*_ is the interface stress between the liquid and solid; *γ*_*l*_ is the liquid surface tension; *θ* is the cusp angle; and *φ* is the contact angle (Supplementary information). The surface stress of pristine CY52-276 that is obtained with glycerol is 42.8 ± 1.4 mN/m, which is close to the value 42 mN/m measured by Park *et al.*^41^. The values of the surface stress of the pristine PDMS that are obtained with glycerol and liquid paraffin are 34.1 ± 1.6 mN/m and 31.8 ± 1.2 mN/m, respectively, in good agreement with each other. The surface stress of one layer graphene-covered PDMS which is obtained with glycerol is 58.4 ± 1.1 mN/m, whereas that obtained with liquid paraffin is 60.6 ± 3.1 mN/m. These values are consistent, and significantly larger than the value of the corresponding bare substrate.

### Model of deformation with surface stress of solid

We use the extracted surface stress in the model developed above to describe the deformation profile. Assuming *γ*_*s*_ = *γ*_*sl*_, the linearized tensile stress *σ*_*zz*,*γ*_(*r*,0) in the vertical direction can be related to the vertical deformation *v*(*r*,0) as^3^,^43^,^44^


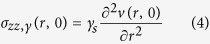


**Figure 4:**
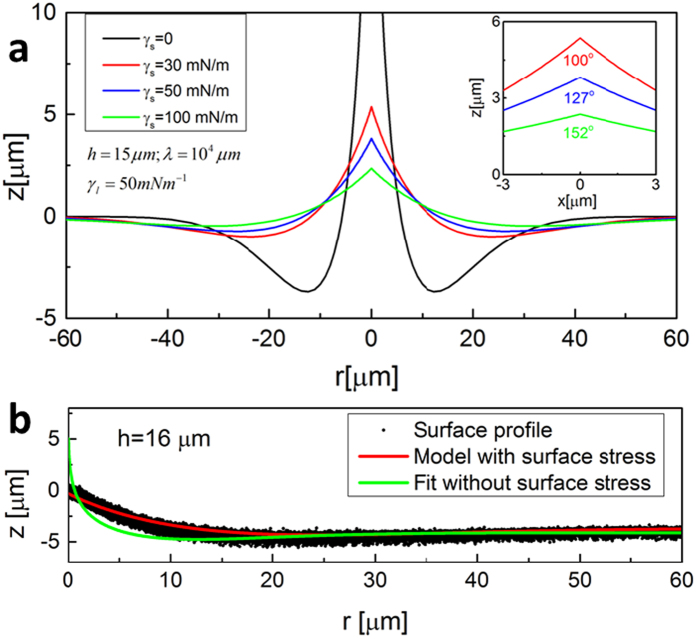
(**a**) Deformation profile of the same substrate as Fig. 3 but incorporating different values of surface stress *γ*_*s*_. The insert is close-up of the cusp region. The fitted cusp angle increases with increasing *γ*_*s*_. (**b**) Best fittings of theoretical models with and without surface stress of the solid are superimposed on the experimental data obtained from a glycerol droplet laying on a 16 μm thick silicone substrate covered by one layer of graphene.

This problem can be solved analytically (Supplementary information). Figure 4 shows the profiles of the deformed surfaces calculated from the above theory including the surface stress. The singularity of the displacement at the contact line is removed when considering the effect of the surface stress (Fig. 4a), as for pure silicone^43^. The theory also gives a region near the contact line whose profile can be fitted by a cusp with an open angle (the insert of Fig. 4a). Figure 4b displays the measured deformation profile induced by a glycerol droplet standing on a substrate covered by one layer of graphene (scattered black dots), and the analytical solutions with (red line) and without (green line) the surface stress. The theoretical model incorporating the measured surface stress provides a good quantitative description of the out-of-plane deformation of the graphene-covered substrate, as found for pure substrates^43^.

### Surface stress of single layer graphene and multilayer graphene supported on substrate

**Figure 5:**
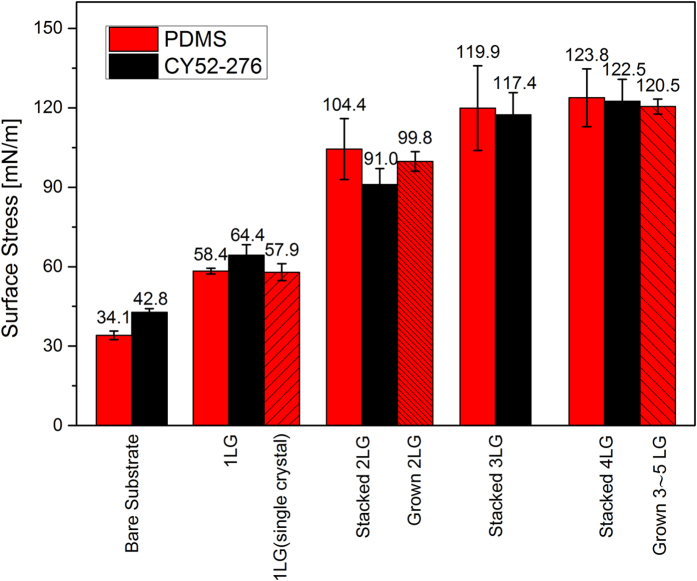
Surface stress of substrates covered by single layer graphene and multilayer graphene.

The single layer graphene we used above is polycrystal at the millimeter scale and it contains grain boundaries. Using the same method, we measure the surface stress of a centimeter-sized single crystal of a single layer of graphene^50^. Shown in Fig. 5, the surface stress of the single crystal graphene supported on PDMS is 57.9 ± 3.2 mN/m, which is very close to 58.4 ± 1.1 mN/m of the polycrystal graphene. This behaviour is similar to the finding that the stiffness of polycrystal graphene is close to that of single crystal graphene^47^.

Next, we study the surface stress of multilayer graphene supported on a substrate. Here multilayers are obtained by two methods. The first one is the widely used layer-by-layer transfer method, which can precisely control the layers of graphene and is referred to as “stacked” graphene in Fig. 5. The second method is direct transfer of grown multilayer graphene (referred to as “grown” in Fig. 5), which would guarantee the standard Bernal stacking of graphene. The interface stresses at the graphene-silicone interface and between the graphene layers are found well below the interface strengths. Thus, the graphene layers will neither separate from the substrate, nor from each other, in the experiments. Figure 5 shows the extracted surface stress of substrates covered by different numbers of layers of graphene. The surface stress of the stacked multilayer graphene supported on PDMS is comparable with that of the grown multilayer graphene for the cases of two layers and 3∼5 layers. Increasing graphene layers enhances the surface stress, and finally the surface stress reaches an asymptotic value when the substrate is covered by three layers of graphene. The surface stress of multilayer graphene is thickness-dependent, which is similar to the surface energy of graphene that is related to the substrate and thickness^51^,^52^. The asymptotic values of the surface stress of the multilayer graphene supported on PDMS and CY52-276 are consistent and are about 120 mN/m measured with glycerol.

## Discussion

In this study, we extracted the surface stress using the method developed in ref. 40 which assumes that the cusp orientation is symmetric and perpendicular to the surface of the substrate. Recently, by using the X-ray microscopy, Park *et al.*^41^ found that, in general, the cusp induced by the surface stress at the three-phase contact line may be bent to form an asymmetric tip. Based on the geometry of the cusp measured by X-ray microscopy, Park *et al.*^41^ found that the surface stress of CY52-276 is 42 mN/m, which is close to the value 42.8 ± 1.4 mN/m we obtained with glycerol. On the other hand, Park *et al.*^41^ pointed out that for systems with *γ*_s_ ≈ *γ*_sl_ (that is, φ ≈ 90°), the cusp profile is approximately symmetric, except its tip. Thus, the model of Style *et al.*^40^ may provide a reasonably accurate prediction for the solid surface stress when the contact angle is close to 90°. In our experiments, the graphene-covered substrates have a much stronger resistance to the applied liquid surface tension than bare silicone. The contact angle of glycerol on these substrates is very close to 90° (about 98°, Supplementary information). Therefore, the model of Style *et al.*^40^ can be used to extract the surface stress of the graphene-covered substrates, and the values of the surface stress of the graphene-covered substrates we obtained with glycerol droplets can be deemed to be reasonably accurate. However, the applicability of the experiment and the model is based on the condition that the layered system can support the applied force while still keeping the interface well bonded, which is true in this study. If the interface bonding is very weak such that debonding or sliding takes place under the action of the droplet, the experiment and the model will not be applicable.

The surface stress reaches a constant value on three and more layers of graphene means that the surface stress is mainly contributed by the three atomic layers near the surface, which constitutes a direct experimental confirmation of the general theoretical result that the contribution to the surface stress is mainly from the surface atomic layer and two layers under it^2^. Nevertheless, the increase of the surface stress with increasing layers of graphene on the substrate demonstrates that the substrate plays an important role.

In conclusion, by measuring the deformation of substrates induced by the surface tension of liquid droplets, we find that the graphene-covered soft substrates have similar deformation profiles to that of the bare substrate, while the deformation magnitude is significantly reduced. The theoretical model that includes the effect of surface stress can remove the singularity of the deformation and thus agrees with the experimental measurement. Based on the Neumann’s triangle concept, we extract the surface stress of bare and graphene-covered substrates. The apparent surface stress of one layer graphene supported on the silicone substrate (PDMS) is 58.4 ± 1.1 mN/m, measured with glycerol, and is 60.6 ± 3.1 mN/m obtained with liquid paraffin. This apparent surface stress increases with the number of the graphene layers, and eventually reaches a value of ∼120 mN/m on three and more layers of graphene. The results reveal that the contribution to the surface stress of multilayer graphene is mainly from the three layers in the vicinity of the surface. This work provides a basic parameter of supported multilayer graphene, which is the commonly used structure in graphene-based devices^21^,^22^,^23^,^24^,^31^,^32^,^33^. The method may also be used to measure the surface stress of other ultrathin films supported on soft substrates.

## Methods

### Fabrication of silicone substrate

Two kinds of soft silicone, i.e., polydimethylsiloxane (PDMS, Sylgard 184, Dow Corning) and CY52-276 (Dow Corning), were used in our experiments. The soft PDMS samples were prepared by spin-coating the oligomer with a base-curing ratio 75:1 on clean smooth glass slides for 1 min at different speeds (1000, 3000 and 5000 rpm). After curing at 75 °C for 6 hours, we obtained soft silicone substrates of different thicknesses (70 μm, 30 μm and 16 μm). The estimated Young’s modulus of the gel is ~2 kPa^53^,^54^. Following a procedure similar to that reported by Style *et al.*^16^, we fabricated another soft silicone substrate of CY52-276. The Young’s modulus of this kind of gel is ~3 kPa^16^. As PDMS is mainly used in this study, in the Article and in the Supplementary information, soft silicone refers to PDMS-based soft silicone unless CY52-276 is explicitly mentioned.

To obtain the topography of the substrates, we physically attached fluorescent nanobeads (carboxylated Yellow-Green Fluospheres of 200 nm diameter, Invitrogen) to the substrates. Each 180 μm × 180 μm square field of view contained ~2000 fluorescent beads, which covered a total area fraction of no more than 0.002. For PDMS surfaces fabricated by spin coating, the root-mean-square roughness is always less than 2 nm^55^, and should have little effect on the accuracy of the measured deformation.

### Transfer of grapheme

The graphene samples we used were from three sources: the commercially available polycrystal single layer graphene, chemical vapor deposition (CVD) multilayer graphene (two layers and 3~5 layers) on a copper foil (ACS Materials, USA), and single crystal single layer CVD graphene on copper that was provided by the research group of Dr Dong Wang of the Institute of Chemistry of the Chinese Academy of Sciences, who and his co-workers recently developed a facile atmospheric pressure CVD method to grow centimeter-sized single-crystal graphene on copper foil^50^. The graphene on copper was first cut at a proper size (approximately 2 centimeters). The backside graphene was removed by oxygen plasma (100 w for 3 minutes). To obtain continuous covered graphene, it is important to press a flat copper foil on a flat substrate^56^,^57^,^58^. Then the graphene-covered side of the copper foil was attached to the substrate that had embedded fluorescent beads gently to avoid damaging the soft substrate. The copper foil was etched by ammonium peroxydisulfate with a concentration of 0.5 M for ~ 30 minutes. Then the substrate with graphene coverage was washed in pure water for 3 times.

The multilayer graphene-covered silicone was prepared by two ways. Firstly, for as-grown multilayer graphene, the graphene-covered substrate was obtained by direct transfer of as-grown multilayer graphene onto the silicone substrate as mentioned above. Here, we chose two layer as-grown graphene and 3~5 layer as-grown graphene (Characterization of graphene is shown in the Supplementary information) in our experiment. Secondly, single layers of graphene were transferred onto a copper foil layer-by-layer, following the standard wetting transfer procedure^59^. Subsequently, the silicone substrate was attached to the copper foil covered by the multilayer graphene stack, and the copper was etched by the same procedure as above.

In the Article and in the Supplementary information, the single layer graphene will refer to the polycrystal single layer graphene that was purchased from ACS Materials (USA) unless an explicit statement is made to the single crystal single layer graphene of Li *et al.*^50^.

### Measurement of deformation profiles

We followed the method in ref. 40. A spinning disk confocal microscope (PerkinElmer, mounted on a Nikon Ti Eclipse inverted microscope with an oil immersion 40X objective and numerical aperture (NA) = 1.3) was employed to image the beads. We used two kinds of liquid droplet, glycerol and liquid paraffin, to induce the deformation. Three-dimensional image stacks of the fluorescent tracer particles were acquired less than 20 seconds after a liquid droplet became stabilized on a substrate. The positions of the nanobeads were then extracted by Gaussian fits and azimuthally collapsed to radial surface profiles under each droplet.

## Additional Information

**How to cite this article**: Du, F. *et al.* Surface stress of graphene layers supported on soft substrate. *Sci. Rep.* **6**, 25653; doi: 10.1038/srep25653 (2016).

## Supplementary Material

Supplementary Information
