# Supplementary material for: Surface stress of graphene layers supported on soft substrate
Source: Sci Rep. 2016 May 11;6:25653. doi: 10.1038/srep25653 (PMC4863371; doi:10.1038/srep25653)
Supplement: Supplementary Information [file srep25653-s1.docx]

Surface stress of graphene layers supported on soft substrate

Feng Du^1^, Jianyong Huang^1, 4^, Huiling Duan^1, 2^, Chunyang Xiong^1, 3, *^, Jianxiang Wang^1, 2, *^

^1^State Key Laboratory for Turbulence and Complex System, and Department of Mechanics and Engineering Science, College of Engineering, Peking University, Beijing 100871, China

^2^CAPT-HEDPS, and IFSA Collaborative Innovation Center of MoE, Peking University, Beijing 100871, China

^3^Academy for Advanced Interdisciplinary Studies, Peking University, Beijing 100871, China

^4^Department of Biomedical Engineering, Duke University, Durham, NC, 27708, USA

*To whom correspondence should be addressed:

*Chunyang Xiong, Ph.D. Department of Mechanics and Engineering Science, College of Engineering, Peking University, Beijing 100871, China

Email: [cyxiong@pku.edu.cn](mailto:cyxiong@pku.edu.cn); Phone: 86-10-6275-7940

*Jianxiang Wang, Ph.D. Department of Mechanics and Engineering Science, College of Engineering, Peking University, Beijing 100871, China

Email: [jxwang@pku.edu.cn](mailto:jxwang@pku.edu.cn); Phone: 86-10-6275-7948

**Supplementary information**

1. Experimental methods
   1. Characterization of graphene

Raman spectra were carried out using a LabRAM ARAMIS System (HORIBA Scientific) equipped with a 532 nm laser source. The laser spot has a diameter of 1 μm. To avoid laser induced heating and damage to graphene, the laser power was kept below 2 mW during scan. The surface morphology of transferred graphene was investigated by atomic force microscope (AFM, MFP-3D, Asylum Research) utilizing the tapping mode with a silicon cantilever of spring constant 1.2 N/m and resonance frequency 75 KHz.

- 1. Measurement of contact angle

Static contact angle measurements on bare substrates and graphene-covered ones were performed at room temperature (22-25 °C) and a relative humidity of 27-43%. An automatic contact angle meter (OCA 20, DataPhysics, Germany, precision ±0.1°) was used in the experiments. Glycerol and liquid paraffin were used as the test liquids. A volume of 2 microliter drop was used during the contact angle measurements. Each group contained 3 samples, and measurements were performed at 3 randomly chosen test points on each sample. All the results are mean values plus standard deviations.

- 1. Measurement of liquid surface tension

The surface tension of the wetting liquids was determined by the hanging droplet technique. First, a liquid droplet as large as possible was hung on the automatic contact angle meter (OCA 20, DataPhysics, Germany). Then, photographs of the hanging droplet were taken after it became stabilized. The liquid tension was extracted from the shape of the hanging droplet using the method developed in ref. [1]. At least 5 droplets were tested for each wetting liquid.

1. Experimental results
   1. Raman, AFM and optical microscope characterization of graphene

We used the Raman shift technique [2, 3] to analyze the characteristics of Raman peaks, and the AFM to scan the surface morphology at the microscopic scale. A normal Raman spectrum of a graphene sample usually contains three peaks D, G and 2D. As shown in Figure S1(a) for a single layer of graphene transferred onto a silicone substrate, the position of the G peak was at 1588 cm^-1^ while the 2D peak was at 2675 cm^-1^ with its full width half-maximum (FWHM) being 30 cm^-1^. The integrated intensity ratio of 2D to G was found to be larger than 2, whereas the D peak was very small, which implies that the graphene on silicone was a single layer with few defects in nature [2, 3]. Figure S1(b) shows the AFM image of one layer graphene on silicone. From it, we find that the graphene sample has a homogenous and crack free surface.

In order to evaluate the quality of graphene at a relatively large scale, optical microscope was used to characterize the coverage of graphene after being transferred onto soft silicone. Results in Figure S2(a) show a homogenous brightness except some dark spots, which may be the aggregated fluorescence beads that were used to track the substrate deformation. This homogenous brightness implies that the graphene layer is homogenous and crack free at the scale of tens of micrometers. Figure S2(b) shows the region where a continuous graphene film was torn into several micrometer-scale patches, with the cracks easily recognized, which was attributed to the light adsorption of graphene. In order to certify that the homogenous brightness character indeed represents homogeneity and integrity of the graphene layer, we stretched the graphene covered silicone along the horizontal direction to produce graphene cracks. Figure S2(c) shows the stretched graphene on a silicone sample with an elongation of about 10%. From it, we can easily find that several relatively bright stripes exist on the stretched sample and they are primarily along the vertical direction. Because of the light adsorption of graphene, these bright stripes should be the pure silicone, which implies that the relatively dark homogenous region is a graphene-covered region. Based on the optical microscope character of graphene on silicone shown in Figure S2, we can easily choose the high quality and integrate graphene-covered region to conduct the deformation test subsequently.

The coverage of a single crystal of graphene was also probed by optical microscopy and shown in Figure S3. Figure S3(a) shows the photograph of single crystal patches of graphene which are transferred onto a polymer base of polyvinyl butyral (PVB) to facilitate observation. From it, we can easily find that the nucleation points of graphene are randomly scattered and the graphene patches have a hexagonal shape except some intersected ones. This hexagonal shape of graphene patches are consistent with the results reported by Li et al.[4], which demonstrates that these patches are in fact single crystals of single layer graphene, as characterized and demonstrated by Li et al.[4]. The size of the single crystal graphene patches is in the scale of centimeter or several millimeters. The optical microscopy of a transferred single crystal graphene on the silicone substrate is shown in Figure S3(b). The transferred graphene has a homogenous brightness. The homogenous brightness indicates that the graphene is continuous and integrate. Figure S3(c) shows the optical microscopy of the boundary of a single crystal graphene patch. The graphene-covered area can be easily identified and this patch has a corner angle of about 120°. All these characteristics demonstrate that the transferred single crystal graphene of a single layer has a homogenous coverage and high integrity at the scale of at least several millimeters, which can guarantee the measurement of the property of single crystal graphene on the soft substrate by the droplet.

The Raman characters of the transferred as-grown multilayer graphene are shown in Figure S4. Figure S4(a) shows the Raman spectrum of the as-grown two layer graphene. From it, we find that the G peak is higher than the 2D peak and the 2D peak lies at 2690 cm^-1^, with its FWHM about 44 cm^-1^. These characters imply that the graphene has a thickness of 2 layers [5]. It should be mentioned that the current technology to grow multilayer graphene on copper can accurately control the number of layers when the layers are less than or equal to two layers [6], as confirmed by the above case of two layers. When the number of layers is three or more, the technology cannot control the exactly required number of layers, but can give a range of the layers, and the range can be verified by the Raman spectrum. Figure S4(b) shows the Raman spectrum of the as-grown 3~5 layer graphene sample, where the 2D peak lies at 2700 cm^-1^ and its FWHM is about 49 cm^-1^, which illustrates that the thickness of graphene is more than 3 layer [5]. All these characters demonstrate that the transferred as-grown multilayer graphene has a thickness that is consistent with the specification of the product.


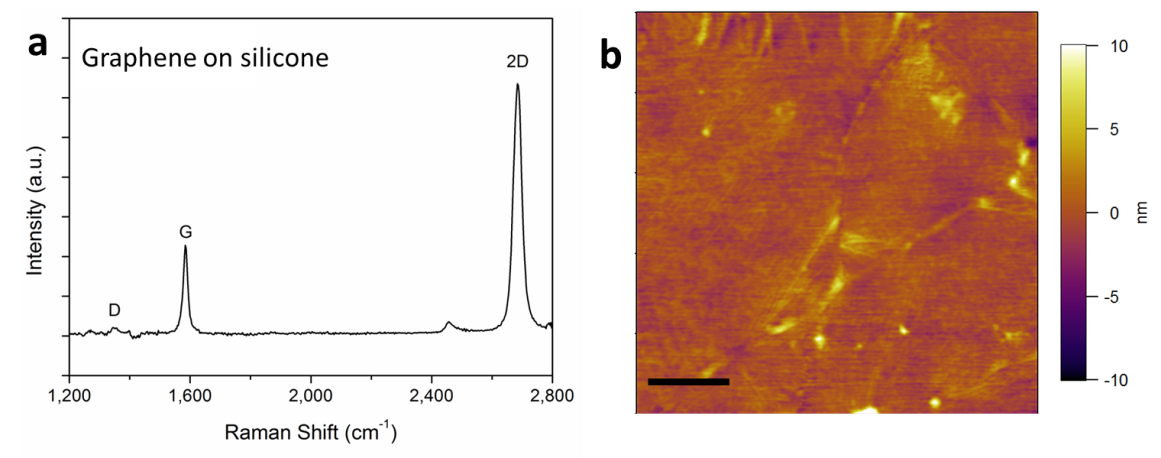


Figure S1. Raman and AFM characterization of single layer graphene. (a) Raman spectrum of single layer graphene on silicone. (b) AFM image of single layer graphene on silicone. Scale bar is 1 μm.


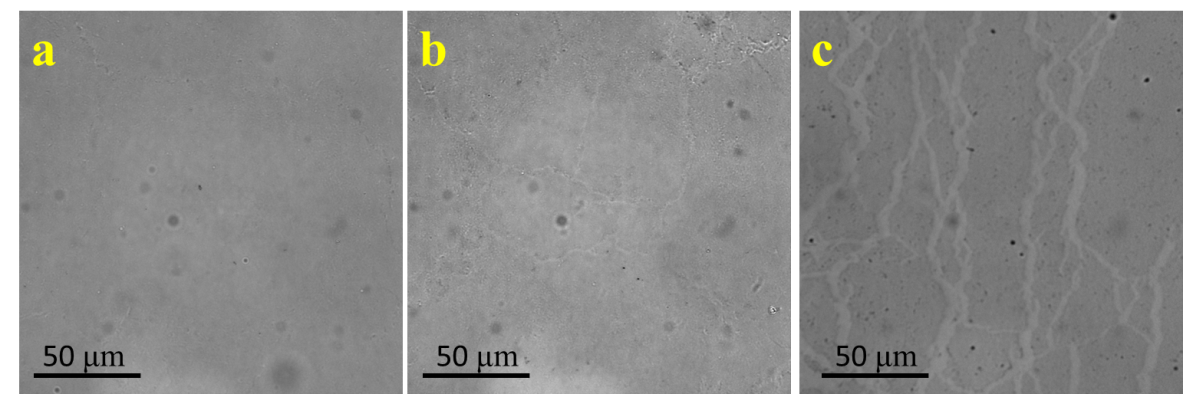


Figure S2. Optical microscope characterization of polycrystal graphene. (a) Optical microscopic image of single layer graphene on silicone with a crack free region. (b) Optical microscopic image of single layer graphene on silicone with several cracking regions. (c) Optical microscopic image of single layer graphene on silicone under stretching state. The sample was stretched along the horizontal direction and the elongation is about 10%.


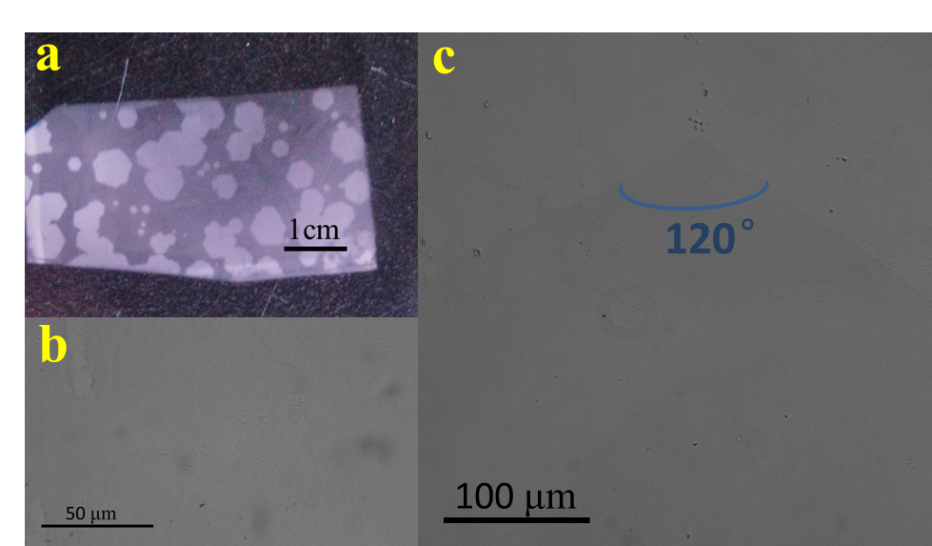


Figure S3. Optical microscopic characterization of single crystal graphene. (a) Photograph of graphene patches on a polymer substrate. (b) Optical microscopic image of a single layer of single crystal graphene on silicone. (c) Optical microscopic image of the boundary of a single crystal graphene patch.


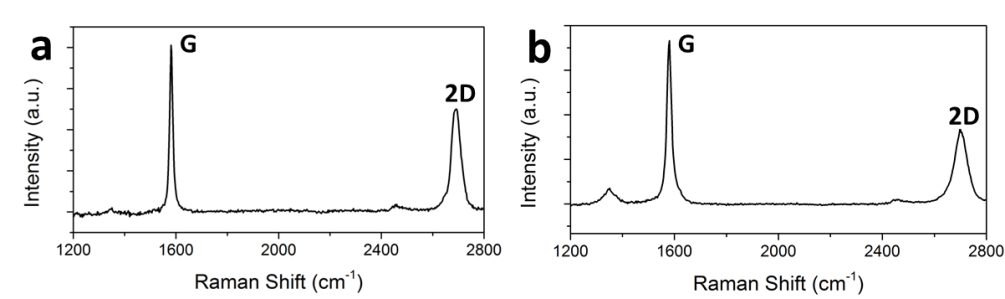


Figure S4. Raman characterization of transferred as-grown multilayer graphene. (a) Raman spectrum of transferred as-grown two layers of graphene. (b) Raman spectrum of transferred as-grown 3~5 layers of graphene.

- 1. Deformation profile induced by liquid paraffin droplet on bare and one layer graphene-covered substrate

Figure S5 shows the deformation profiles of PDMS substrates of different thicknesses with and without being covered by one layer of graphene, where the deformation is induced by the surface tension of liquid paraffin droplets.

Figure S6 shows the vertical displacement that is caused by the three phase contact line on 30 µm thick PDMS substrates with and without being covered by one layer of graphene.

Figure S7 shows the open angles of the cusps on a bare PDMS substrate and a substrate covered by one layer of graphene.


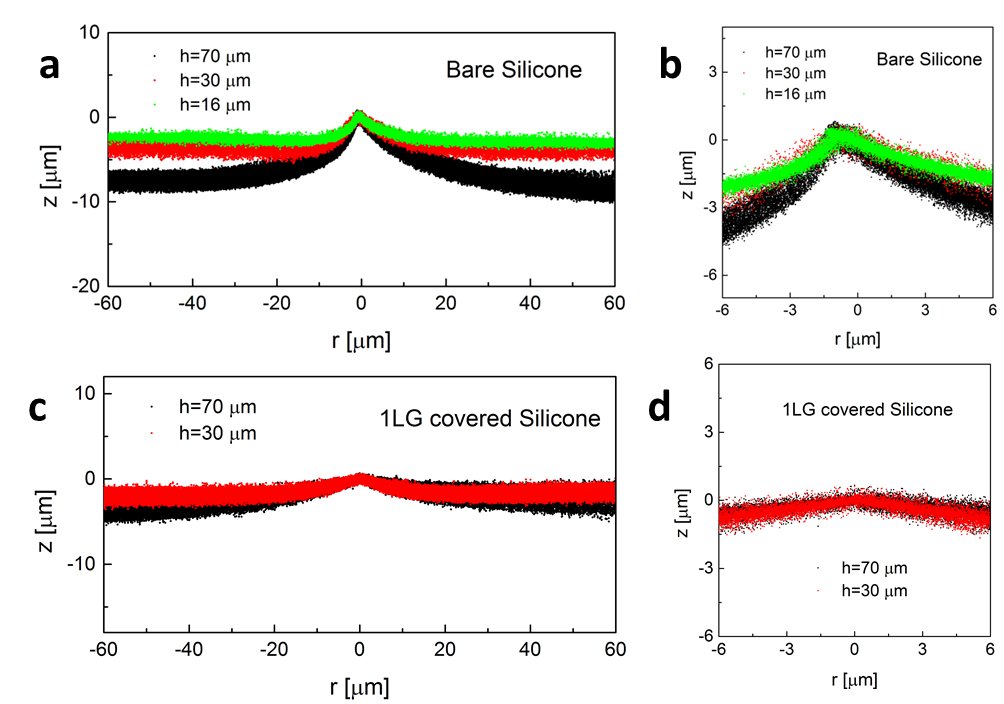


Figure S5. Deformation profiles (a) and close-ups (b) caused by liquid paraffin droplets on PDMS substrates with different thicknesses. Deformation profiles (c) and close-ups (d) caused by liquid paraffin droplets on PDMS substrates with different thicknesses that are covered by one layer of graphene (1LG covered silicone).


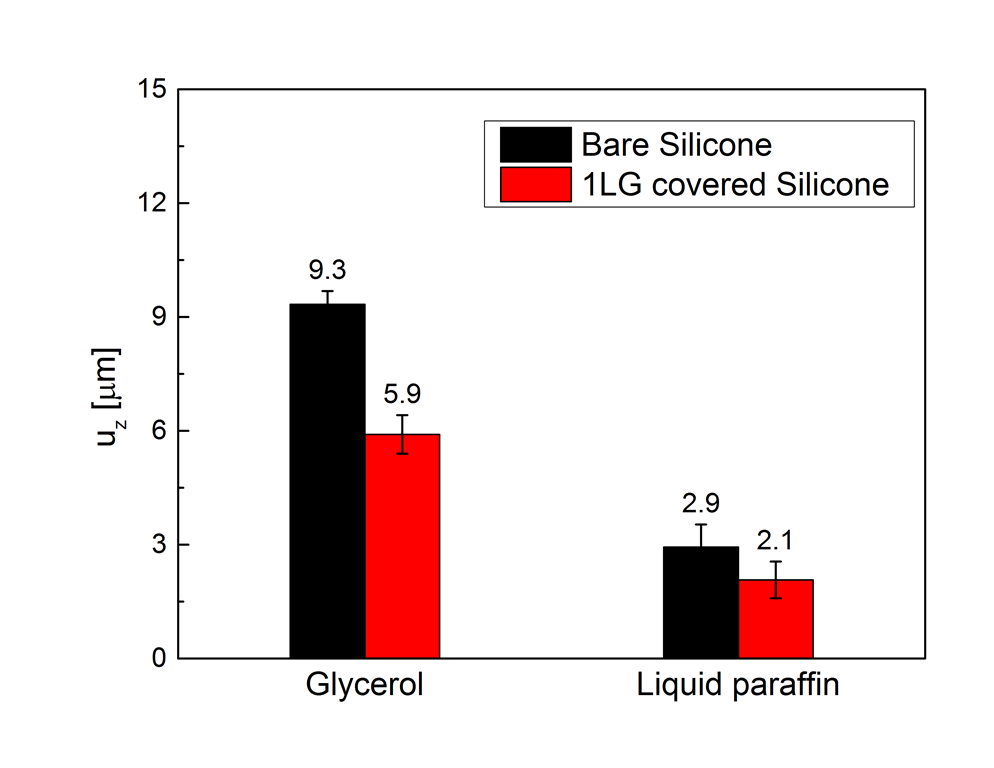


Figure S6. Vertical displacements caused by the three phase contact line on 30 µm thick PDMS substrates with and without being covered by one layer of graphene.


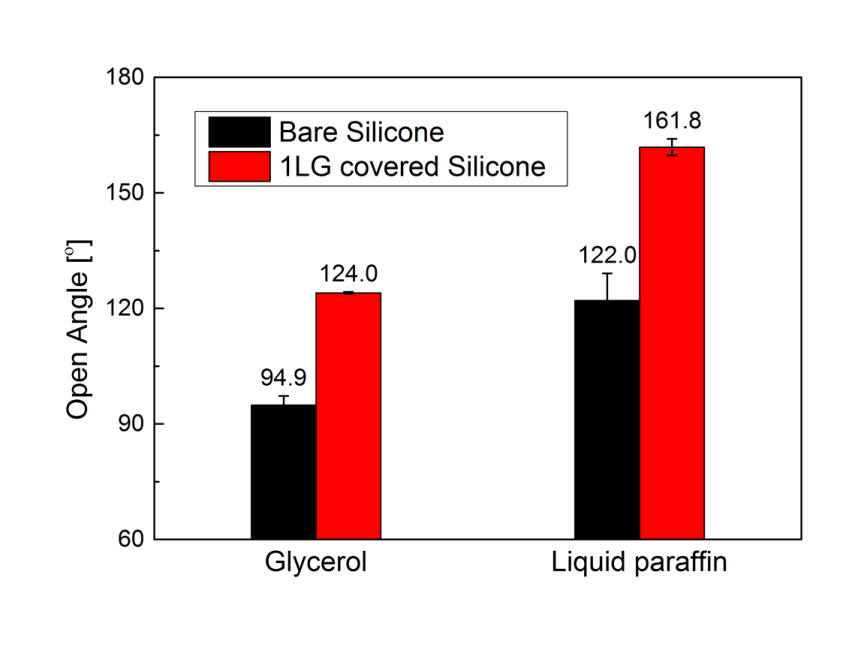


Figure S7. Open angles of cusps near the contact line of different probe liquids on bare PDMS substrates and ones covered by one layer of graphene.

- 1. Open angles of cusps induced by glycerol on substrates covered by multilayer graphene

Figure S8 shows the open angles of cusps induced by glycerol on PDMS substrates covered by multilayer graphene.


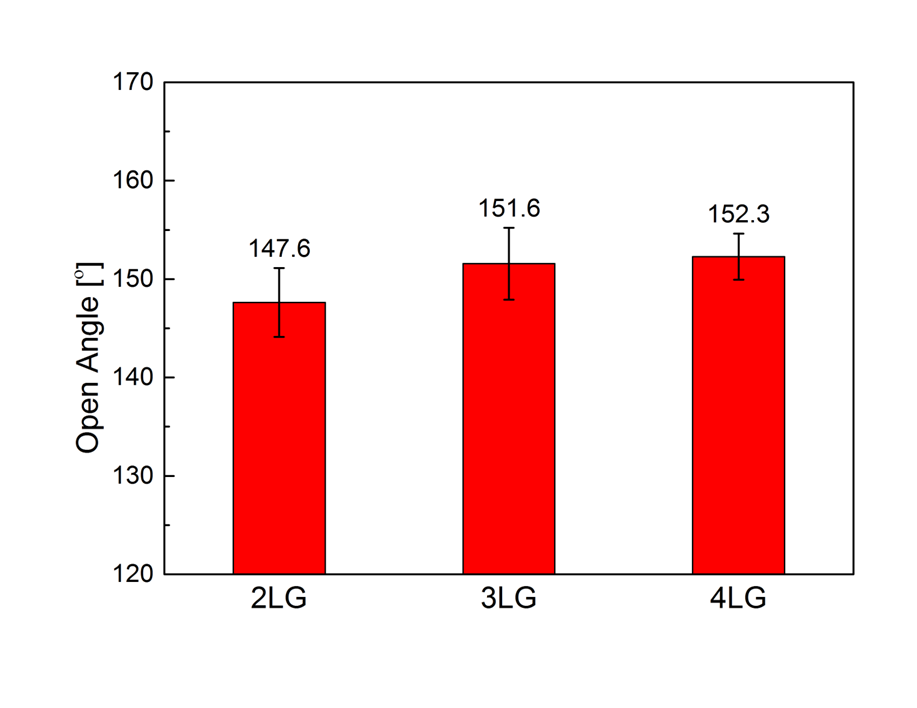


Figure S8. Open angles of cusps induced by glycerol on PDMS substrates covered by two, three and four layers of graphene.

- 1. Contact angles

Figure S9 shows the contact angles of glycerol and liquid paraffin on bare and graphene-covered PDMS substrates. The contact angle test is conducted in ambient environment, and the contact angle may be affected by the joint effect of both graphene and environmental adsorbates [7].


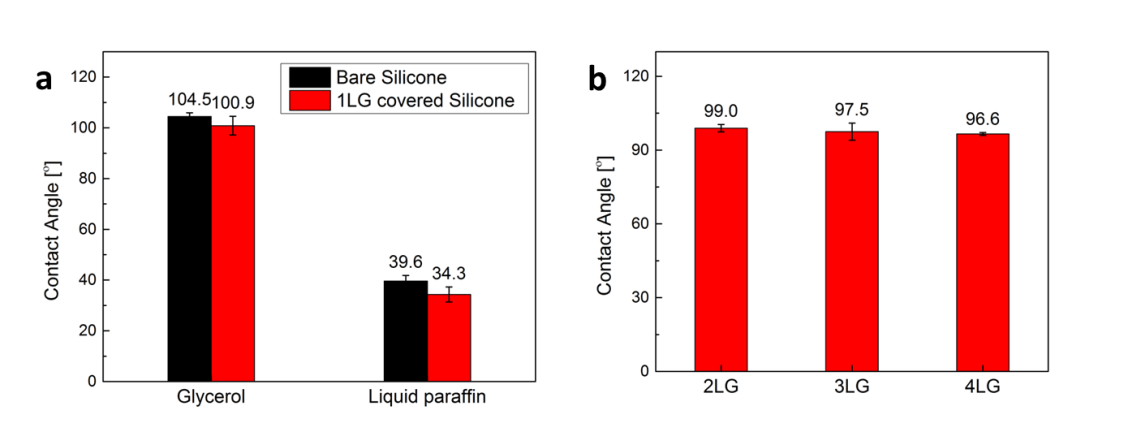


Figure S9. Contact angles of glycerol and liquid paraffin on bare PDMS substrates and substrates covered by one layer of graphene (a). Contact angles of glycerol droplets on multilayer graphene-covered substrates (b). The contact angle decreases with increasing graphene layers.

- 1. Liquid surface tension

Figure S10 shows the liquid surface tension of fresh glycerol, liquid paraffin and glycerol that contact with a silicone substrate.


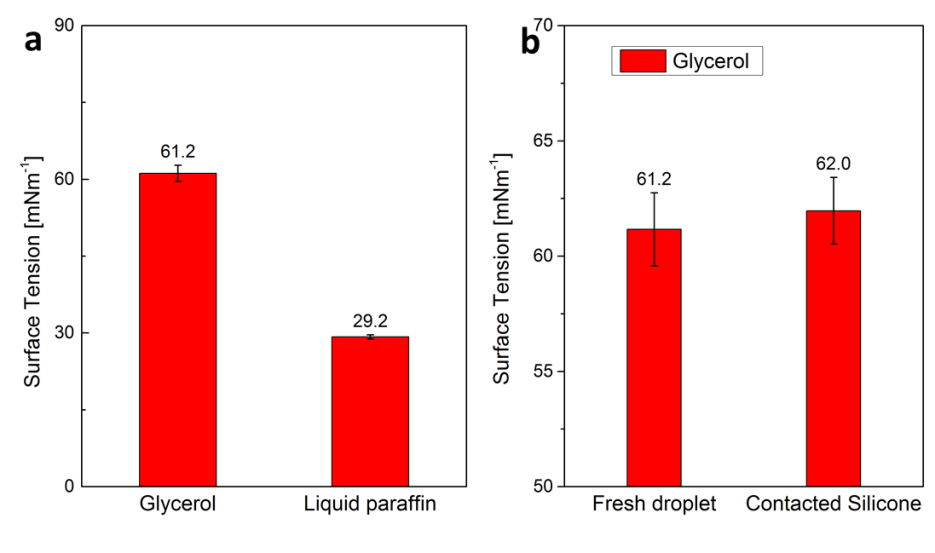


Figure S10. Liquid surface tension of glycerol and liquid paraffin in their fresh state (a). Liquid surface tension of glycerol that contacts with a PDMS substrate (b). The contact of glycerol with the PDMS substrate does not influence the surface tension of glycerol noticeably.

1. Details of theoretical model


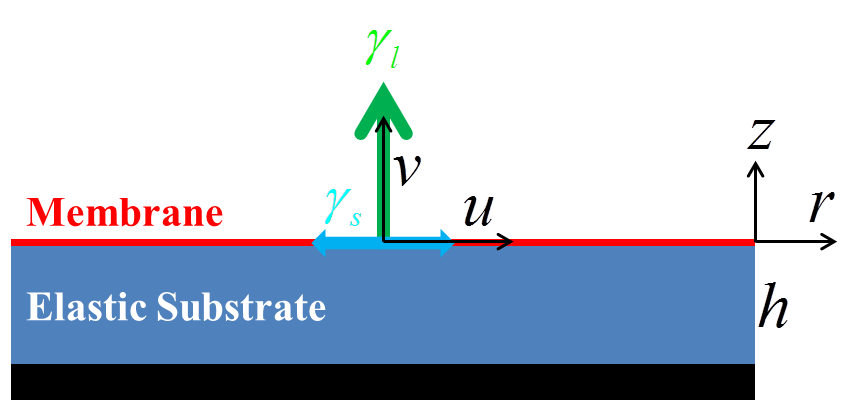


Figure S11. Schema of the model.

The thickness of the silicone substrate is at the scale of micrometers, while the contact radius of a liquid droplet on the substrate in our experiment is larger than 2 millimeters, so the deformation of the substrate can be approximately described by a two-dimensional elastomer with its lower boundary fixed (schema shown in Fig. S11) [8]. Graphene is modeled as a membrane for its thickness is ultra-small compared to other length scales of the system. Based on the Airy stress function theory [9], the stress components $\sigma_{ij}$ of the substrate can be related to the Airy stress function $\phi\left( r,z \right)$ as

$\left\{ \begin{aligned} \sigma_{rr}=\frac{\partial^{2}\phi}{\partial z^{2}} \\ \sigma_{zz}=\frac{\partial^{2}\phi}{\partial r^{2}} \\ \sigma_{rz}=-\frac{\partial^{2}\phi}{\partial r\partial z} \end{aligned} \right.$ (1)

where$r$and $z$ represent the radial and vertical coordinates from the position of the contact line on the surface of the substrate, respectively. The stress function $\phi$should satisfy the biharmonic equation

$\nabla^{2}\phi=\left( \frac{\partial^{2}}{\partial r^{2}}+\frac{\partial^{2}}{\partial z^{2}} \right)\left( \frac{\partial^{2}}{\partial r^{2}}+\frac{\partial^{2}}{\partial z^{2}} \right)\phi=0$ (2)

When the deformation is small, from the elastic constitutive relation of an isotropic material, the in-plane deformation$u\left( r,0 \right)$of the membrane can be related to its in-plane stress$\sigma_{rr}\left( r,0 \right)$by [10]

$\sigma_{rr}\left( r,0 \right)=A_{g}\varepsilon_{rr}=A_{g}\frac{\partial u\left( r,0 \right)}{\partial r}$ (3)

where$A_{g}=\frac{E_{g}}{1-\upsilon_{g}^{2}}$, and$E_{g}$, $\upsilon_{g}$are the Young’s modulus and Poisson’s ratio of the membrane, respectively. The upper boundary of the membrane is free and the lower boundary is fixed with the substrate, so the equilibrium equation of the membrane can be expressed as

$h_{g}\frac{\partial\sigma_{rr}\left( r,0 \right)}{\partial r}=-q\left( r \right)$ (4)

where$h_{g}$is the thickness of the membrane, and $q\left( r \right)$is the interface stress between the membrane and the substrate. From equations (3) and (4), the deformation of the membrane can be related to the interface stress by

$\frac{E_{g}h_{g}}{1-\upsilon_{g}^{2}}\frac{\partial^{2}u\left( r,0 \right)}{\partial r^{2}}=-q\left( r \right)$ (5)

To introduce the effect of the surface stress, assuming that the surface stress of the solid is equal to the interface stress between the liquid and solid, which is valid for an equilibrium contact angle of 90^0^, the linearized stress in the vertical direction$\sigma_{zz,\gamma}$can be related to the solid surface stress$\gamma_{s}$and the vertical deformation $v\left( r,0 \right)$by [8, 11, 12]

$\sigma_{zz,\gamma}\left( r,0 \right)=\gamma_{s}\kappa=\gamma_{s}\frac{\partial^{2}v\left( r,0 \right)}{\partial r^{2}}$ (6)

where$\kappa$ is the curvature, which is approximated by the second-order derivative of the vertical displacement.

The deformation of the substrate is constrained by four boundary conditions. First, the force in the vertical direction follows the equilibrium equation

$\sigma_{zz}\left( r,0 \right)+\sigma_{zz,\gamma}\left( r,0 \right)=-p\left( r \right)$ (7)

where$p\left( r \right)$is the applied vertical stress, and here we apply a unit concentrated force at the origin. Second, the interface stress between the membrane and substrate follows

$q\left( r \right)=-\sigma_{rz}\left( r,0 \right)$ (8)

The last two are the rigid boundary conditions at the lower interface

$\left\{ \begin{aligned} u\left( r,-h \right)=0 \\ v\left( r,-h \right)=0 \end{aligned} \right.$ (9)

where$h$ is the thickness of the soft substrate.

To obtain an analytical solution of this problem, the Fourier transform method is used to determine the stress function. The biharmonic equation (2) is

$\frac{\partial^{4}G}{\partial z^{4}}-2w^{2}\frac{\partial^{2}G}{\partial z^{2}}+w^{4}G^{4}=0$ (10)

where$G\left( w,z \right)$is the Fourier transform of$\phi$. The general solution of equation (10) is

$G\left( w,z \right)=\left( A+Bz \right)e^{-wz}+\left( C+Dz \right)e^{wz}$ (11)

where the constants$A,B,C,D$ are functions of $w$and to be determined by the four boundary conditions, which can be written as

$\left\{ \begin{aligned} w^{2}G\left( w,0 \right)+\lambda_{s}\left[ \frac{\partial^{3}}{\partial z^{3}}G\left( w,0 \right)-3w^{2}\frac{\partial}{\partial z}G\left( w,0 \right) \right]=1 \\ \lambda\left[ \frac{\partial^{2}}{\partial z^{2}}G\left( w,0 \right)+\frac{\upsilon}{1-\upsilon}w^{2}G\left( w,0 \right) \right]=\frac{\partial}{\partial z}G\left( w,0 \right) \\ \frac{\partial^{2}}{\partial z^{2}}G\left( w,-h \right)+\frac{\upsilon}{1-\upsilon}w^{2}G\left( w,-h \right)=0 \\ \frac{\partial^{3}}{\partial z^{3}}G\left( w,-h \right)-\frac{2-\upsilon}{1-\upsilon}w^{2}\frac{\partial}{\partial z}G\left( w,-h \right)=0 \end{aligned} \right.$ (12)

where$\lambda_{s}=\frac{\gamma_{s}\left( 1-\upsilon^{2} \right)}{E}$is the modified elasto-capillary length of$\frac{\gamma_{s}}{E}$; $E$and$\upsilon$ are the Young’s modulus and Poisson’s ratio of the substrate, respectively; and$\lambda=\frac{E_{g}h_{g}}{E}\frac{1-\upsilon^{2}}{1-\upsilon_{g}^{2}}$ is the equivalent thickness of the membrane. Inserting equation (11) into (12), the four constants $A,B,C,D$can be determined uniquely.

In the Fourier space, the vertical displacement$v\left( r,0 \right)$at the upper boundary of the substrate is

$\hat{v}\left( w,0 \right)=\frac{3}{2Ew}\frac{\left[ -1+2w\lambda+e^{4hw}\left( 1+2w\lambda\right)-4e^{2hw}w\left( h+\lambda+2h^{2}w^{2}\lambda\right) \right]}{\left[ \begin{aligned} \left[ 1-2w\lambda+e^{4hw}\left( 1+2w\lambda\right)+e^{2hw}\left( 2+4h^{2}w^{2}+8hw^{2}\lambda\right) \right] \\ +2w\lambda_{s}\left[ -1+2w\lambda+e^{4hw}\left( 1+2w\lambda\right)-4e^{2hw}w\left( h+\lambda+2h^{2}w^{2}\lambda\right) \right] \end{aligned} \right]}$ (13)

When the membrane does not exist, we set$\lambda=0$, and then equation (13) can be simplified to be

$\hat{v}_{\lambda=0}\left( w,0 \right)=\frac{3}{2Ew}\frac{\left[ -1+e^{4hw}-4e^{2hw}hw \right]}{\left[ 1+e^{4hw}+e^{2hw}\left( 2+4h^{2}w^{2} \right) \right]-2w\lambda_{s}\left[ 1-e^{4hw}+4e^{2hw}hw \right]}$ (14)

which is the same as that in ref. [8]. When ignoring the surface stresseffect, i.e., setting$\lambda_{s}=0$, equation (13) becomes

$\hat{v}_{\lambda_{s}=0}\left( w,0 \right)=\frac{3}{2Ew}\frac{\left[ -1+2w\lambda+e^{4hw}\left( 1+2w\lambda\right)-4e^{2hw}w\left( h+\lambda+2h^{2}w^{2}\lambda\right) \right]}{\left[ 1-2w\lambda+e^{4hw}\left( 1+2w\lambda\right)+e^{2hw}\left( 2+4h^{2}w^{2}+8hw^{2}\lambda\right) \right]}$ (15)

This means that $\hat{v}_{\lambda_{s}=0}\left( w,0 \right)$scales as $\frac{3}{2Ew}$ when$w\to\infty$, which implies that the vertical displacement is singular (i.e. tends to infinity) at the origin no matter how stiffness the membrane is.

Based on the above derivation, the horizontaland vertical displacements of the upper side of the substrate can be expressed as

$$\left\{ \begin{aligned} v\left( r,0 \right)=\frac{3}{4\pi E}\int_{-\infty}^{+\infty} \frac{\left[ -1+2w\lambda+e^{4hw}\left( 1+2w\lambda\right)-4e^{2hw}w\left( h+\lambda+2h^{2}w^{2}\lambda\right) \right]\cos\left( wr \right)}{\left[ \begin{aligned} \left[ 1-2w\lambda+e^{4hw}\left( 1+2w\lambda\right)+e^{2hw}\left( 2+4h^{2}w^{2}+8hw^{2}\lambda\right) \right] \\ +2w\lambda_{s}\left[ -1+2w\lambda+e^{4hw}\left( 1+2w\lambda\right)-4e^{2hw}w\left( h+\lambda+2h^{2}w^{2}\lambda\right) \right] \end{aligned} \right]}dw \\ u\left( r,0 \right)=\frac{3}{\pi E}\int_{-\infty}^{+\infty} \frac{e^{2hw}h^{2}w\sin\left( wr \right)}{\left[ \begin{aligned} \left[ 1-2w\lambda+e^{4hw}\left( 1+2w\lambda\right)+e^{2hw}\left( 2+4h^{2}w^{2}+8hw^{2}\lambda\right) \right] \\ +2w\lambda_{s}\left[ -1+2w\lambda+e^{4hw}\left( 1+2w\lambda\right)-4e^{2hw}w\left( h+\lambda+2h^{2}w^{2}\lambda\right) \right] \end{aligned} \right]}dw \end{aligned} \right.$$

This is the basic solution of the deformation that is induced by a unit force on the two-dimensional elastic solid substrate of a finite thickness. This solution takes into account the mechanical reinforcement effect of the membrane, and the surface stress. The results shown in Fig 3 in the Article are based on this solution while setting$\lambda_{s}=0$ to exclude the surface stress effect. The results shown in Fig 5 include the mechanical reinforcement effect of the membrane, and the surface stress.

References

1 Fordham, S. On the calculation of surface tension from measurements of pendant drops. *Proc. R. Soc. Lon. Ser-A* **194**, 1-16 (1948).

2 Ferrari, A. C., *et al.* Raman spectrum of graphene and graphene layers. *Phys. Rev. Lett.* **97**, 187401 (2006).

3 Ferrari, A. C. & Basko, D. M. Raman spectroscopy as a versatile tool for studying the properties of graphene. *Nat. Nanotechnol.* **8**, 235-246 ( 2013).

4 Li, J., et al. Facile growth of centimeter-sized single-crystal graphene on copper foil at atmospheric pressure. *J. Mater. Chem. C***3**, 3530-3535 (2015).

5 Wang, Y., *et al.* Interface engineering of layer-by-layer stacked graphene anodes for high-performance organic solar cells. *Adv. Mater.* **23**, 1514-8 (2011).

6 Lee, S., Lee, K. & Zhong, Z.H., Wafer scale homogeneous bilayer graphene films by chemical vapor deposition. *Nano Lett.* **10**, 4702-4707 (2010).

7 Kozbial, A., et al., Study on the surface energy of graphene by contact angle measurements. *Langmuir* **30,** 8598-8606 (2014).

8 Jerison, E. R., Xu, Y., Wilen, L. A. & Dufresne, E. R. Deformation of an elastic substrate by a three-phase contact line. *Phys. Rev. Lett.* **106**,186103 (2011).

9 Gupta, P. K. & Walowit, J. A. Contact stresses between an elastic cylinder and a layered elastic solid. *J. Lubric. Tech.-T. Asme* **96**, 250-257 (1974).

10 King, R. B. & Osullivan, T. C. Sliding contact stresses in a two-dimensional layered elastic half-space. *Int. J. Solids Struct.* **23**, 581-597 (1987).

11 Style, R. W. & Dufresne, E. R. Static wetting on deformable substrates, from liquids to soft solids. *Soft Matter* **8**, 7177-7184 (2012).

12 Cammarata, R. C. & Sieradzki, K. Surface and interface stresses. *Annu. Rev. Mater. Sci.* **24**, 215-234 (1994).
